# Dynamical backaction cooling with free electrons

**DOI:** 10.1038/ncomms9104

**Published:** 2015-09-18

**Authors:** A. Niguès, A. Siria, P. Verlot

**Affiliations:** 1 Laboratoire de Physique Statistique de l'Ecole Normale Supérieure, UMR8550, ENS, 24, rue Lhomond, 75005 Paris, France; 2 Institut Lumière Matière, UMR5306 Université Lyon 1-CNRS, Université de Lyon, 69622 Villeurbanne Cedex, France

## Abstract

The ability to cool single ions, atomic ensembles, and more recently macroscopic degrees of freedom down to the quantum ground state has generated considerable progress and perspectives in fundamental and technological science. These major advances have been essentially obtained by coupling mechanical motion to a resonant electromagnetic degree of freedom in what is generally known as laser cooling. Here, we experimentally demonstrate the first self-induced coherent cooling mechanism that is not mediated by an electromagnetic resonance. Using a focused electron beam, we report a 50-fold reduction of the motional temperature of a nanowire. Our result primarily relies on the sub-nanometre confinement of the electron beam and generalizes to any delayed and spatially confined interaction, with important consequences for near-field microscopy and fundamental nanoscale dissipation mechanisms.

## Abstract (web summary)

Cooling atoms and ions to the quantum ground state is generally achieved by resonantly coupling their mechanical motion to an electromagnetic wave. Here the authors report self-induced cooling based on sub-nanometre confinement with an electron beam, rather than an electromagnetic resonance.

## Graphical abstract

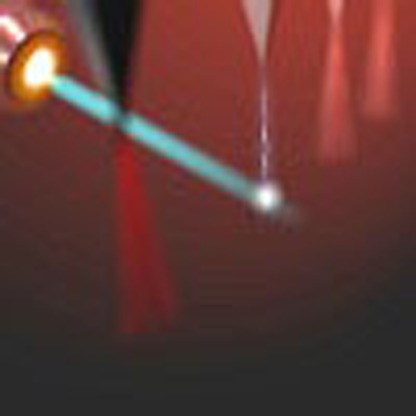

Coherent manipulation of mechanical motion is one of the great challenges of Modern Physics^1^: driven by such outstanding goals as reaching the zero-point motion fluctuations of single quantum objects^2^,^3^, observing the quantum behaviour of large atomic ensembles^4^,^5^,^6^, or engineering systems for quantum information processing^7^, a number of efficient schemes have been proposed and implemented^8^,^9^,^10^,^11^, establishing laser control as a paradigm for cooling and trapping matter at the microscopic scale^12^. In recent years, the field of cavity optomechanics has demonstrated that this paradigm extends remarkably well to the macroscopic level^13^,^14^, with demonstrations of radiation-pressure-induced cooling^15^,^16^ down to the quantum ground state^17^,^18^.

Despite a considerable variability both in concepts and experimental realizations, all the above cited experiments relied on the fundamental interactions between a mechanical degree of freedom and an electromagnetic resonance (for example, atomic transitions^19^, Fabry–Perot resonance^20^, two-level systems^21^,^22^,^23^) that collects the mechanical energy.

In this work, we report the first dynamical backaction cooling experiment that is not mediated by an electromagnetic resonance. We demonstrate that under the illumination of a continuous focused electron beam, a nanowire can spontaneously reach an equilibrium with drastically reduced motional temperature. We develop a simple and general model and attribute this behaviour to the presence of dissipative force gradients generated by the electron-nanowire interaction. From a general perspective, our results point out the potential of spatial confinement to provide the same functions as optical confinement in laser cooling, with important consequences for interpreting and controlling near-field dynamics at the nanoscale^24^,^25^,^26^,^27^. Moreover, the dramatic spatial dependence of the effectively measured mechanical damping rate emphasizes the prominent importance of taking into account the spatial environment for explaining dissipation mechanisms at the nanoscale, whose fundamental limits remain an opened question^28^,^29^,^30^,^31^,^32^. In a more specific scope, our work shows that electron microscopy is perfectly suited to ultra-sensitive, perturbation-free dynamical studies at the nanoscale, with performances comparable to laser sensing^33^,^34^, however with a 100 times higher confinement. This represents a very attractive perspective for sensitive investigation of mono-dimensional structures dynamics such as carbon nanotubes^35^ and graphene^36^. Last, on a more technical side, our results show that electron microscopy intrinsically holds the ability to suppress the unavoidable thermal vibrations of nano-structures, yielding to a significant improvement of the image resolution.

## Results

### Detecting nanoscale dynamics with a SEM

**Figure 1:**
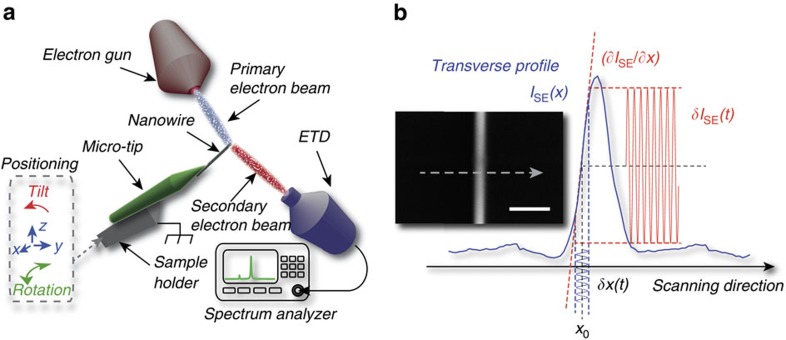
Detecting nanoscale dynamics with a SEM. (**a**) Schematic of the experimental set-up. The nanowire is mounted onto the three-dimensional-positioning platform of a commercial SEM, which includes an electron gun delivering a stable collimated flux of electrons, and a SEs detector (Everhart–Thornley Detector (ETD)), whose output is used for both imaging the nanowire and measuring its dynamical motion around its equilibrium position. (**b**) Using Secondary Emission for nanomechanical motion detection. The very high contrast of SEM imaging (illustrated here with a 20 nm gold nanowire, scale bar, 200 nm) results in a highly peaked evolution of the SE rate as a function of the transverse displacement. The nanomechanical motion *δx* around its equilibrium position *x*_0_ is therefore transduced into large variation of the SE emission rate.

The nano object of interest in this work consists of a cylindrical Silicon Carbide (SiC) nanowire with length *L*=150 μm and diameter *d*=250 nm. The nanowire is glued at the edge of a Tungsten micro-tip sitting on an Aluminium sample holder. The ensemble is mounted in vacuum onto the (grounded) three-dimensional-positioning stage hosted in a commercial scanning electron microscope (SEM; NOVA NANOSEM, FEI), see Fig. 1a. Scanning electron microscopy provides an image of the surface of a given sample through its response to a focused beam of electrons. The collisions between the incident electrons and the sample yield to a variety of interaction products, including light, X-rays and electrons, which can be further detected and used for imaging purposes^37^. In this study, we will focus our interest on the so-called secondary electrons (SEs), which are ejected from the sample owing to strongly inelastic collisions between the primary electron beam and the surface of the target. These electrons are guided to a dedicated detector (Everhart–Thornley Detector), which includes a strongly biased grid and a high bandwidth scintillator. Importantly, secondary emission is an absorption sensitive mechanism and therefore captures both relief and composition, making SE response the most used imaging mode in SEM.

Here we turn secondary emission from its conventional use and show that it intrinsically holds additional capabilities for sensitive dynamical studies^38^. The idea is depicted in Fig. 1b: scanning an individual nano object in a given direction *x*, its presence is revealed under the form of a sharp peak. Its nanomechanical displacements *δx* around its rest position *x*_0_ will therefore result in large variations of the SE emission rate 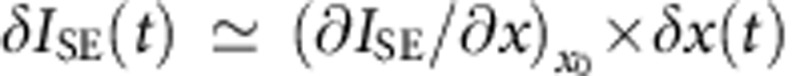, with *I*_SE_(*x*) denoting the average SE emission rate as a function of position *x*. The efficiency of such a scheme is primarily determined by the SE response contrast, which is typically high for a wide class of nano-objects and materials^39^.

**Figure 2:**
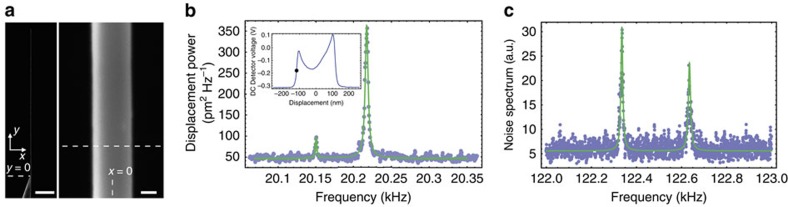
Brownian motion detection of a SiC nanowire in a SEM. (**a**) SEM static images of the nanowire used in the present study. The magnification coefficients are × 1,500 (left, scale bar, 20 μm) and × 250,000 (right, scale bar, 100 nm). The nanowire is mounted into the horizontal plane (*x*,*y*), with respective origins on the nanowire axis (right) and at the apex of the Tungsten micro-tip (left). (**b**) Calibrated ETD noise spectrum 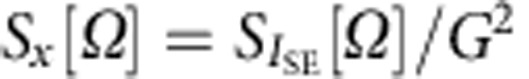 acquired in spot mode, with the electron probe being set at *y*_0_=10 μm. Inset shows a line scan taken at the same longitudinal distance, and which served for determining the local slope 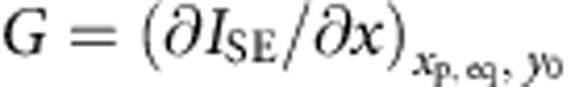. The dark dot indicates the transverse position at which the spectrum was acquired. (**c**) Brownian motion spectrum associated with the second harmonic vibration around *Ω*/2*π*≃122.5 kHz.

We have used this principle for sensitive motion detection and characterization of the SiC nanowire introduced above. All the measurements presented hereafter have been obtained with an incident electron beam current *I*^in^=140 pA and an accelerating voltage *V*=5 kV, corresponding to an incident power *P*^in^=0.7 μW. Figure 2a shows two SEM images (with magnification coefficients of × 1,500 and × 250,000, respectively). A very high contrast can be observed, suggesting a very efficient motional transduction into the SE emission rate. To further verify this assertion, we turn the SEM into ‘spot mode' operation. We set the primary electron beam at position (*x*_0_=−100 nm, *y*_0_=10 μm) (*x* and *y* denote the transverse and longitudinal coordinates with respective origins taken on the axis of the nanowire and at its clamping point, see Fig. 2a). The SE emission rate is collected via the real-time detector output of the SEM and further sent to a spectrum analyser. Two peaks are found around a frequency *Ω*/2*π*≃20 kHz (see Fig. 2b), in agreement with the theoretically expected fundamental resonance frequency 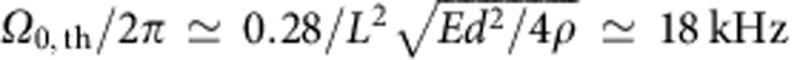 (SiC density *ρ*≃3,000 kgm^−3^, and Young's modulus *E*≃400 GPa). We verified the presence of a pair of resonances around *Ω*/2*π*≃120 kHz (Fig. 2c), corresponding to approximately six times the fundamental resonance frequency, and thereby completing the series of eigenmodes associated with a free-standing cantilever beam. We have also calibrated the measured spectrum into an equivalent transverse displacement (Fig. 2b). This is accomplished by dividing the measured fluctuations by the static transduction factor, that is, the slope 
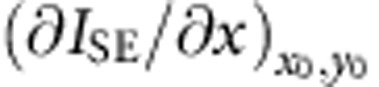
 inferred from the line scan (inset, Fig. 2b). This calibration yields a displacement variance Δ*x*^2^=(68 pm)^2^. It is worth to compare this value to the thermal variance expected for such nanowire, 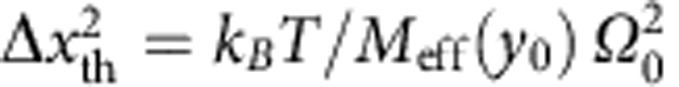, with *k*_*B*_ Boltzmann's constant, *T*=300 K the ambient temperature, and 
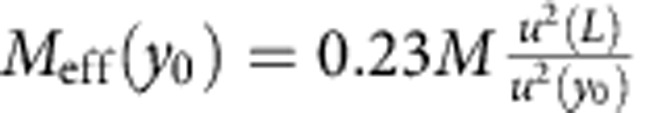
 the effective mass^40^ (*u* being the mode shape function associated with the fundamental flexural mode of the nanowire and *M* the physical mass of the nanowire, *M*=*ρ* × *πd*^2^*L*/4). For *y*_0_=10 μm, we find 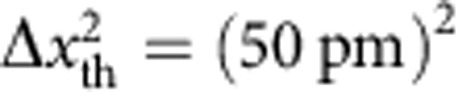, in very good agreement with the above calibrated value. We will henceforth assume the nanowire to be in contact with an external thermal bath with constant temperature *T*=300 K. Last, we have also measured spectra at various tilt angles (not shown), resulting in an effective rotation of the nanowire vibrational axis with respect to the horizontal plane. We have thus verified that the relative heights of both peaks seen in Fig. 2b could be changed and even inverted, which we have used to match both the scanning and vibrational planes. In the following, we concentrate on the dynamical evolution of the in-plane vibration and will neglect the contribution of the out-of-plane mode to the measured nanomechanical spectrum.

### Dynamics in retarded force gradients

**Figure 3:**
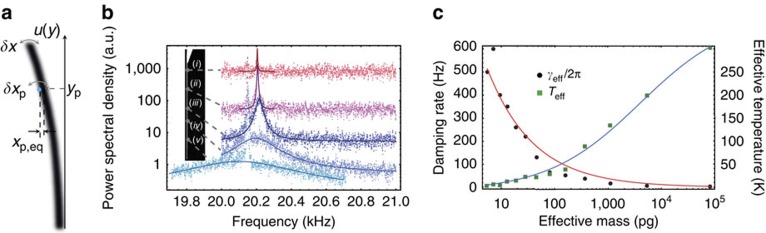
Dynamical backaction cooling with electrons. (**a**) Schematic introducing the notations used in the text. The electron probe (blue spot) is at the average position (*x*_p,eq_, *y*_p_). The dynamical displacement *δx*_p_ around the position of the probe and the tip displacement *δx* are related via the mode shape function *u* of the nanowire. (**b**) Dynamical backaction cooling using an electron beam. The longitudinal position of the electron spot is scanned across the entire nanowire length (left), with the transverse coordinate *x*_p,eq_ being fixed. For each point, the corresponding fluctuation spectrum is recorded (acquisition time ≃2 min). The presented data are normalized to the same effective mass, to better visualize the drastic decrease of the effective temperature. Straight lines are a double Lorentzian adjustment of the experimental data. We attribute the observed amplification of the out-of-plane mode (left most peak) to the presence of orthogonal gradients. (**c**) Effective temperature (green squares) and effective damping rate (black dots) as functions of the effective mass. The straight lines correspond to plots of our theoretical model (equation 4), yielding *Γ*_e_(*x*_p,eq_)/2*π*=604 Hz.

The above reported thermal noise spectrum has been obtained within unfavourable measurement conditions, since the primary electron beam was coupled to the nanowire close to its anchor point, where its effective mass is very large (here *M*_eff_ (*y*_0_=10 μm)=130 ng). In a next step, we have therefore moved the electron probe towards the edge of the nanowire, expecting a rapid increase of the signal-to-noise ratio as a function of the longitudinal displacement *y*. Surprisingly, this is not what we observed: instead, the signal-to-noise ratio remained rather constant, whereas the spectral width of the transverse mode was dramatically increased (see Fig. 3b).

The displacements of the nanowire are governed by the general dynamical equation:


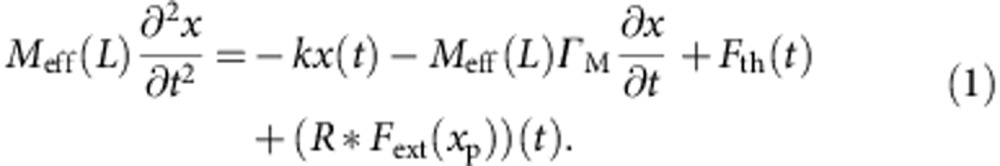


Here *x* denotes the transverse displacement of the nanowire, *k* its lateral spring constant, *Γ*_M_ its intrinsic damping rate, *F*_th_(*t*) the thermal Langevin force (with spectral density 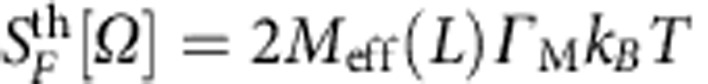), *F*_ext_ the static external force field in which the nanowire is moving and *x*_p_ the time-dependent point of application of the force. The last term in equation (1) takes into account the external force changes *Ω* resulting from the nanomechanical motion^34^, including some possible retardation effects through a time response *R*(*t*). Keeping only the time-varying component of the displacements *δx* and *δx*_p_ and assuming that they remain small compared with the spatial variations of the external force, it is straightforward to expand equation (1) in Fourier space to obtain:


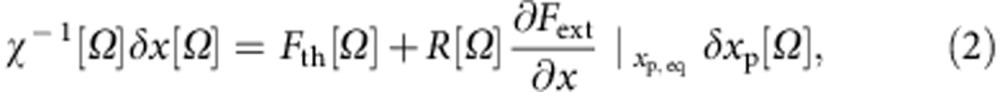


where *Ω* is the Fourier frequency, 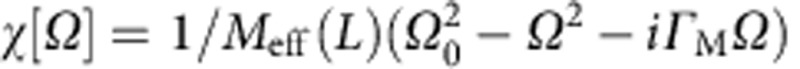 is the mechanical susceptibility associated with the fundamental transverse vibration and *x*_p,eq_ the static displacement of the nanowire at the point of application. In our case, the dominant external force being applied to the nanowire is generated by the primary electron beam, with point of application (*x*_p,eq_, *y*_p_) (see notations in Fig. 3a). The displacement at the point of application are related to the displacement *δx* through the mode shape function *u*, 
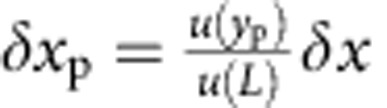
, such that the equation of motion writes in Fourier space *δx*[*Ω*]=*χ*_eff_ [*Ω*]*F*_th_ [*Ω*], with *χ*_eff_ given by:


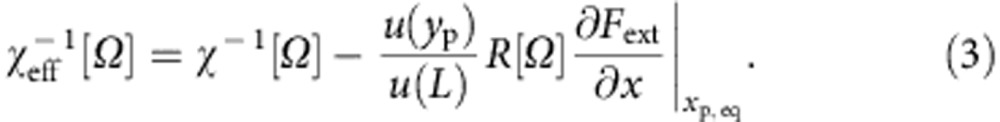


Assuming that the spectral variations of *R* are negligible around frequency *Ω*_0_, equation (3) shows that our system is indeed expected to respond similarly to cavity optomechanical systems^13^, whose effective mechanical response is changed in presence of cavity-delayed optical force gradients. Such delays result in an additional imaginary contribution to the effective susceptibility (the cold damping term^41^, that would be equivalent to the imaginary part of *R*), which manifests as a change of the effective damping rate, and yields to the ability to control the dynamical state of the mechanical resonator.

As already noted above, we observe a very large increase of the effective dissipation when moving the probe towards the extremity of the nanowire, as shown in Fig. 3b. This suggests that important delays are involved into the dynamical interaction between the primary electron beam and the nanomechanical oscillator. This led us to identify the nature of this interaction as being of a thermal origin^42^: when the electron beam hits the sample, a fraction of its energy is released into heat, as a consequence of inelastic mechanisms. To excite the acoustic phonon associated with the fundamental transverse vibrational mode, the produced heat needs to propagate over the entire length^26^,^43^. Here we have assumed a first order low-pass model 
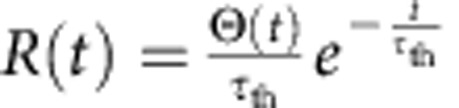
, with Θ the Heaviside step function, *τ*_th_=*L*^2^*ρc*_p_/*κ* the heat diffusion time (*c*_*p*_ the specific heat capacity and *κ* the thermal conductivity). For SiC nanowires, typical values are on the order of *c*_p_≃750 JK^−1^ kg^−1^ and *κ*≃10 W K^−1^ m^−1^, the latter being a factor of 10 lower than the bulk value, typically^44^. Hence, we have for the product *Ω*_0_*τ*_th_≃640≫1, which places our system into a situation equivalent to the resolved sideband regime for the electro-thermal backaction^15^,^45^. This means that we can retain the dissipative contribution of the backaction force only, and write the last term of equation (3) as purely imaginary, *i*(*u*(*y*_p_)/*u*(*y*_L_)) × *M*_eff_(*L*)*ΩΓ*_e_(*x*_p,eq_). Finally, our theoretical analysis predicts the evolution of the effective mechanical damping rate *Γ*_eff_ and temperature 
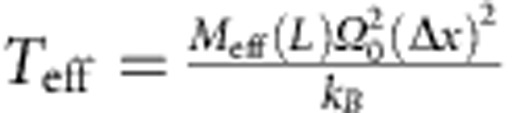
 as a function of the probe longitudinal position:


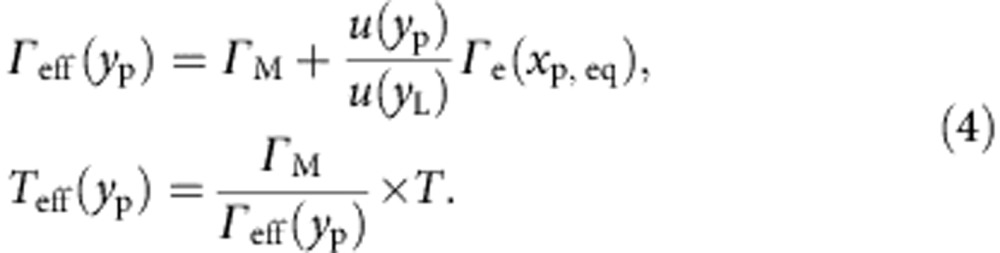


Note that in equation (4), *Γ*_e_, which represents the maximum dissipative coupling strength (when the probe hits the edge of the nanowire), has been assumed to only vary with the transverse degree of freedom *x*_p_ (and not *y*_p_). This is justified because of the very homogeneous SE response of the nanowire (see Fig. 2a), which implies that the electron absorption rate (and hence the resulting thermal force) weakly depends on the longitudinal coordinate. In the low effective damping limit (*Γ*_eff_<<*Ω*_0_) and assuming a long heat diffusion time (*Ω*_0_*τ*_th_≫1), it can be shown that 
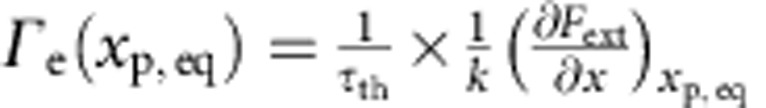
. We also emphasize the peculiar longitudinal dependence of the effective damping 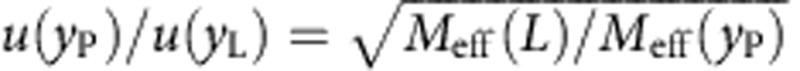, proportional to the inverse square root of the effective mass. This is a signature of the local character of the dynamical backaction, which produces its ponderomotive effects exactly at the point of application, as demonstrated here for the first time.

### Dynamical backaction cooling with a focused electron beam

Figure 3b shows the spectral evolution of the transverse fundamental vibration when moving the probe from the anchor to the edge of the nanowire. The whole set of data were acquired for a value of *x*_p, eq_=−100 nm. The spectra were normalized to the same effective mass, to better represent the dramatic reduction of the effective temperature (proportional to the spectrum area). One can also remark a slight shift of the mechanical resonance frequency at very high gains, characteristic of a residual in-phase contribution (similar to the ‘optical spring') in the dynamical backaction. The experimental data are fitted to a Lorentzian model. The fitting parameters enable to quantitatively determine the longitudinal evolution of both the effective damping rate *Γ*_eff_(*y*_p_) and effective temperature *T*_eff_(*y*_p_), shown in Fig. 3c (black dots and green squares, respectively). Here we chose to report their evolution as functions of the effective mass at the probe location *M*_eff_(*y*_p_), which is a physically more representative parameter. The obtained results are in very good agreement with our theoretical description, as shown by the fitting curves (straight lines, derived from equation (4), with *Γ*_M_/2*π*=4 Hz and *T*=300 K inferred from the reference thermal measurement shown in Fig. 2b) that adjust very well to our experimental data.

**Figure 4:**
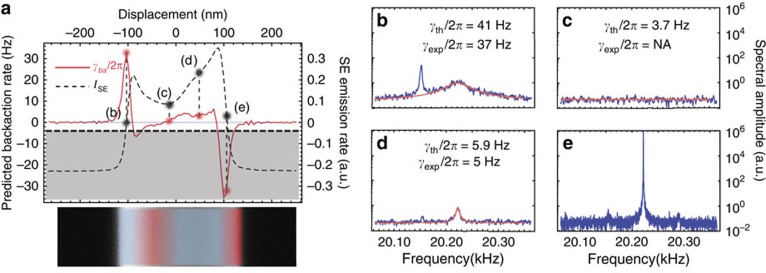
Transverse evolution of the e-beam backaction. (**a**) Line scans of the SE emission rate (dashed, black line) and theoretically expected backaction rate *Γ*_ba_=*Γ*_eff_–*Γ*_M_ (straight, red line) obtained at the longitudinal position *y*_p_=40 μm. The grey zone represents the parametric instability region^13^, where the dynamical backaction cancels the intrinsic mechanical damping rate. Dots with abscissa (**b**–**e**) emphasize the values taken by *I*_SE_ and *Γ*_ba_ at the four acquisition spots. Heating and cooling regions are represented in red and blue on the SEM slice. Right panel: the SE emission rate fluctuation spectrum is measured at transverse positions *x*_p_ (**b**–**e**). The straight lines are Lorentzian adjustments to the experimental data.

To further complete our study, we have also investigated the dynamical backaction effects when moving the equilibrium position of the probe in the transverse direction, for a fixed longitudinal coordinate *y*_p_=40 μm. The corresponding line scan is shown in Fig. 4a (dashed line). As already noted above, SE response reflects the primary electrons absorption rate, which is itself proportional to the force exerted by the electron beam. As a consequence, the strength of the dynamical backaction is expected to be proportional to the gradient of the SE emission rate, (*∂F*_ext_/*∂x*)∝(*∂I*_SE_/*∂x*). Figure 4a shows the theoretically expected backaction rate (straight, red line), obtained by normalizing the line scan derivative to the maximum backaction rate 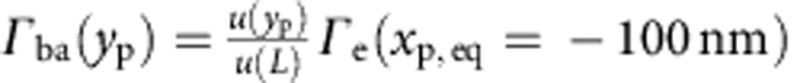 inferred from the measurement presented in Fig. 3c. The right panel of Fig. 4 shows the results obtained at four illustrative transverse positions *x*_p, eq_, labelled from (b–e). The resulting spectra show several interesting features that confirm our theoretical interpretation. Figure 4b,d are both obtained on regions of positive gradient, and are showing motion sensitivities and cooling rates that are proportional to the local slope, in very good agreement with the theoretically expected backaction rates. Figure 4c is obtained with the probe being set on a gradient-free spot, resulting in the total absence of electro-mechanical transduction. Finally, Fig. 4e corresponds to a negative slope, which conversely induces an important amplification of the Brownian motion (beyond the instability threshold, where the dynamical backaction rate cancels the intrinsic damping *Γ*_M_). Importantly, the reversed behaviours observed in Fig. 4b,e reveal the asymmetry of the backaction force with respect to the axis of the nanowire (see the cooling and heating domains in Fig. 4a). This asymmetry reflects that of the force exerted by the electron beam and is a signature of thermally induced bending at the nanoscale^46^,^47^.

## Discussion

Importantly, the ability to cool the motion fluctuations crucially relies on the ultra-low level of fluctuations associated with the cooling pump^41^. For the electron beam, these noises result from the electron shot noise *δI*^in^(*t*), characterized by the white spectral density 
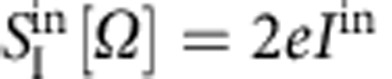
 (*e* the positron charge). These fluctuations generate force noises that eventually limit the cooling efficiency^45^,^48^,^49^,^50^. In general, the force noise associated with any given electron beam-mediated interaction *F*_*k*_ can be written 
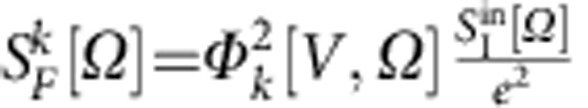
, with *Φ*_*k*_[*V*, *Ω*] denoting the interaction strength per electron per second. Our study gives a quantitative access to this latter quantity in the case of the electro-thermal interaction: From the above given expression of *Γ*_e_, it is straight to obtain 
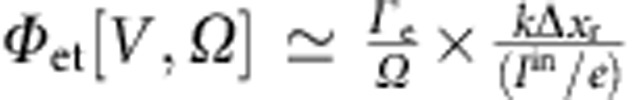
 (Δ*x*_r_ the typical electro-thermal gradient range), where the 1/*Ω* dependence results from high-frequency filtering of electro-thermal fluctuations. Taking Δ*x*_r_=10 nm, *k*=7.9 × 10^−5^ Nm^−1^, and *Γ*_e_/2*π*=604 Hz (as extracted from the fits of the data shown in Fig. 3c), we find *Φ*_et_[*V*, *Ω*]≃2.7 × 10^−23^ N(e^−^)^−1^ s^−1^, and an associated force noise 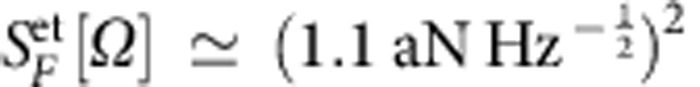. On the other hand, the thermal noise evaluates on the order of 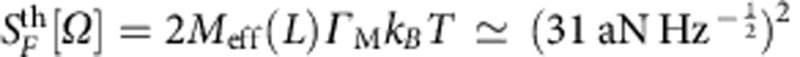: the force noise associated with the electro-thermal backaction is negligible compared with the thermal noise, which defines a cold damping mechanism and justifies that we have neglected it in equation (1).

It is also worth to consider the force noise associated with the ‘electronic pressure' force, resulting from the momentum exchange between the incident electrons and the nanomechanical device. The corresponding interaction strength per electron per second identifies to the average lateral momentum loss per incident electron, *Φ*_*p*_[*V*, *Ω*]=δ*p*_||_(*V*). Accurate evaluation of δ*p*_||_ is a complex problem a priori, requiring advanced simulations of electron transport inside the nanomechanical device^51^. Here we derive a rough estimate by assuming that the single-electron incident lateral momentum noise 
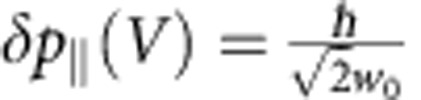
 is integrally transferred to the nanowire (*w*_0_≃1 nm the waist of the electron beam), yielding to *Φ*_*p*_[*V*, *Ω*] ≲7 × 10^−26^ N(e^−^)^−1^ s^−1^, and 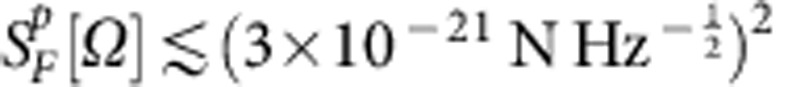. Therefore, the ‘electronic pressure' noise is negligible, the measurement being largely dominated by the quantum electro-thermal noise. This enables to subsequently quantify the quality of the measurement through evaluating the uncertainty products 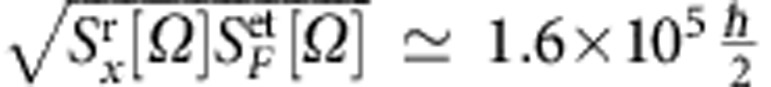 (ref. 22), where we have taken 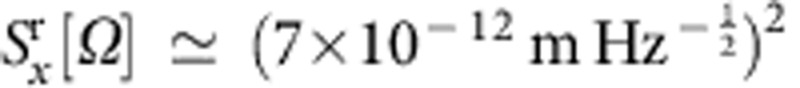 the measurement imprecision inferred from Fig. 2b. Therefore, our measurement scheme operates far above the Heisenberg limit, which is somehow not so surprising, since the measurement and backaction noises are referring to two electronic fields of distinct nature (that is, primary and secondary, respectively). Nevertheless, we note that significant improvement of this limit may be expected by measuring nanomechanical motion via the transmitted electron fluctuations, using for example, a scanning transmission electron microscopy detector.

Interestingly, the efficiency of electron beam cooling scales favourably at higher mechanical resonance frequency. Indeed, the backaction strength can be expressed as a function of the aspect ratio of the nanowire *a*=*L*/*d*, 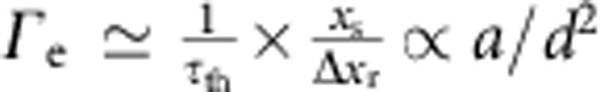, with 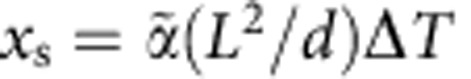 the static nanomechanical displacement^46^ 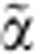 the coefficient of thermal expansion, Δ*T*=*β*_abs_ *R*_th_ *P*^in^ the temperature elevation inside the nanowire, *β*_abs_ its energy absorption coefficient^52^ and *R*_th_=4*L*/*πd*^2^*κ* its thermal resistance. Concurrently, the mechanical resonance frequency scales as *Ω*_0_∝1/*a*^2^*d*: decreasing the diameter *d* while keeping the same aspect ratio therefore yields to both higher frequency and dynamical backaction effects. This perspective is particularly interesting in the context of coherent manipulation of ultra-low phonon number states, since the initial phonon occupancy scales as the inverse of the mechanical resonance frequency *n*_0_=*k*_*B*_*T*/*ħΩ*_0_. Hence, our scheme may provide backaction rates as high as *Γ*_e_/2*π*≃2 MHz for 1-μm long, high aspect ratio nano-structures (*a*≳100), opening the perspective to cool these objects down to their quantum ground state^53^.

In conclusion, we have shown that free electrons establish as an ultra-sensitive, non-invasive probe for measuring and manipulating nanomechanical motion at room temperature. Using a commercial SEM, we have demonstrated that beyond its exquisite static resolution, in the nm range, electron microscopy enables ultra-high, sub-atomic (5 pm range) dynamical sensitivity. We have shown that the SEM appears as an active device that can be used for manipulating the dynamics of a pg-scale nanomechanical device, via an ubiquitous electro-thermal mechanism that creates strong force gradients in the object, while weakly perturbing its static thermodynamic state. In particular, we have used this effect and reported a 50-fold suppression of the transverse vibrational mode thermal energy, representing the first self-induced cooling mechanism that is not mediated by an electromagnetic resonance, but entirely relies on the local force field spatial confinement. Our result therefore appears as a novel, quantitative tool for ultra-sensitive study of electron matter interaction phenomena at nanoscale.

## Methods

### Determination of the effective temperature

For each value of the longitudinal coordinate *y*_p_, the spectrum of the SE emission rate fluctuations 
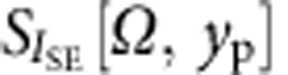
 is recorded together with the local slope 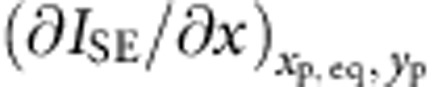, with the transverse coordinate *x*_p,eq_=−100 nm being fixed. Note that as a consequence of the translational invariance of the SE response of the nanowire along the longitudinal direction, the local slope is found to be independent of *y*_p_, 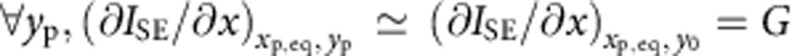. Each spectrum is subsequently multiplied by the local effective mass *M*_eff_ (*y*_p_), yielding a series of spectra *S*_*T*_[*Ω*, *y*_p_] (see Fig. 3b) given by:


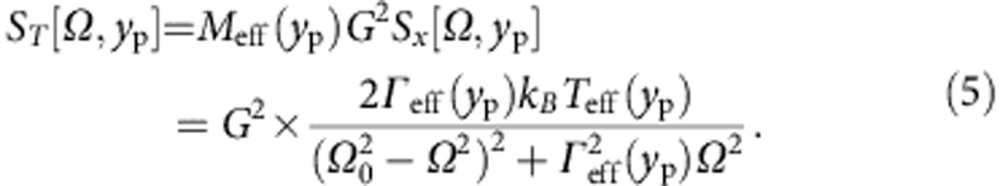


**Figure 5:**
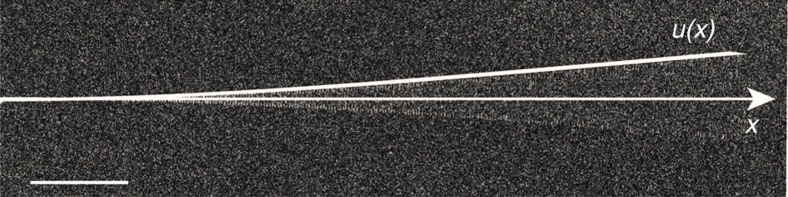
Piezo-driven vibrational profile. A resonant piezo-drive is being applied to the nanowire while scanning its SE response, yielding to a stroboscopic mapping of its fundamental flexural vibrational mode (scale bar, 20 μm). The corresponding profile fits the theoretically expected mode shape given in equation 6 (straight white line), with *β*_1_*L*=1.875 and *L*=150 μm.

The local effective mass is independently measured from a piezo-driven vibrational profile (see Fig. 5), fitting the theoretically expected mode shape *u*(*x*) (straight, white line) given by:


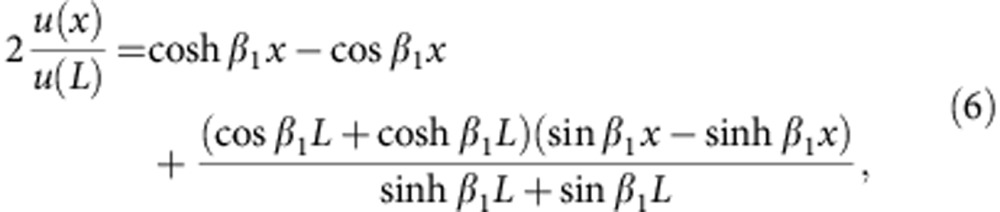


with *β*_1_*L*=1.875 and *L*=150 μm.

The spectra as defined by equation (5) are integrated over the Fourier frequency, yielding in the high-Q limit (*Γ*_eff_<<*Ω*_0_) to a series of quantities proportional to the effective temperature:


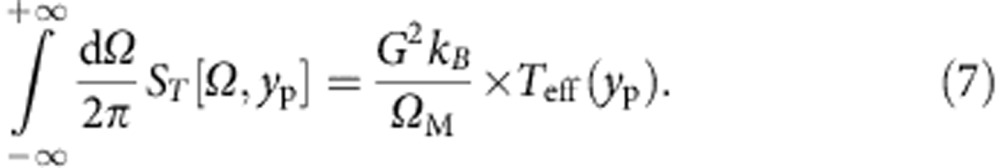


Dividing equation (7) by the area of the reference spectrum *S*_*T*_[*Ω*, *y*_0_] subsequently yields to the cooling rate and equivalently the effective temperature *T*_eff_(*y*_p_),


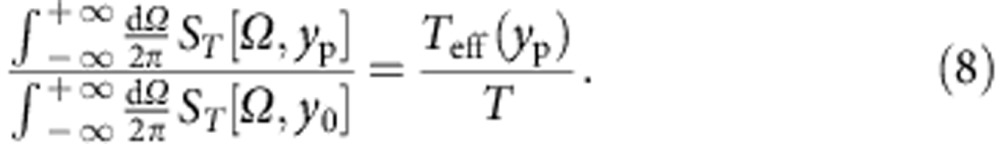


## Additional information

**How to cite this article**: Niguès, A. *et al.* Dynamical backaction cooling with free electrons. *Nat. Commun.* 6:8104 doi: 10.1038/ncomms9104 (2015).
